# Potential Mechanisms of T Cell-Mediated and Eosinophil-Independent Bronchial Hyperresponsiveness

**DOI:** 10.3390/ijms20122980

**Published:** 2019-06-18

**Authors:** Mayumi Saeki, Tomoe Nishimura, Noriko Kitamura, Takachika Hiroi, Akio Mori, Osamu Kaminuma

**Affiliations:** 1 Allergy and Immunology Project, Tokyo Metropolitan Institute of Medical Science, Tokyo 156-8506, Japan; nishimura-tm@igakuken.or.jp (T.N.); kitamura-nr@igakuken.or.jp (N.K.); hiroi-tk@igakuken.or.jp (T.H.); mori-kkr@umin.ac.jp (A.M.); okami@hiroshima-u.ac.jp (O.K.); 2 Clinical Research Center for Allergy and Rheumatology, National Hospital Organization, Sagamihara National Hospital, Kanagawa 252-0392, Japan; 3 Department of Disease Model, Research Institute of Radiation Biology and Medicine, Hiroshima University, Hiroshima 734-0037, Japan; 4 Center for Life Science Research, University of Yamanashi, Yamanashi 400-8510, Japan

**Keywords:** allergy, asthma, airway inflammation, bronchial hyper responsiveness

## Abstract

Bronchial asthma is a chronic disease characterized by reversible airway obstruction, mucus production, and bronchial hyperresponsiveness (BHR). Although Th2 cell-mediated eosinophilic inflammation is an important disease mechanism in the majority of patients with bronchial asthma, recent studies suggest the possible development of Th2-independent airway inflammation and BHR. These non-Th2 endotype patients seem to consist of multiple subgroups, and often do not respond to inhaled corticosteroids. Therefore, to understand the pathogenesis of asthma, it is important to characterize these non-Th2 subgroups. Recently, we demonstrated that Th9 cells induce eosinophil infiltration and eosinophil-independent BHR, and Th9 cells-mediated BHR may be resistant to glucocorticoid. In this review, we summarize the contribution of several T cell subsets in the development of bronchial asthma and introduce our recent study demonstrating Th9 cell-mediated and eosinophil-independent BHR.

## 1. Introduction

There are approximately 330 million individuals with bronchial asthma worldwide [1], with symptoms including episodes of recurrent wheezing, shortness of breath, and chest tightness. Although asthma is considered a complex clinical syndrome whose pathophysiology, severity, natural history, comorbidities, and responsiveness against therapies and drug treatments can vary [2,3], the majority of patients exhibit reversible airway obstruction, mucus production, and bronchial hyperresponsiveness (BHR). Asthma pathogenesis has been widely recognized as allergic, eosinophilic, and Th2-mediated; however, recent analyses of asthmatic patients have suggested clinical phenotypic heterogeneity [4]. Furthermore, a variety of asthma “endotypes” have been described based on different functional or pathophysiological mechanisms [5]. These findings are important for understanding the developmental mechanisms of asthma and producing the next generation of asthma drugs, particularly for chronic and severe cases. Several studies using mouse models of asthma have also suggested the possible involvement of Th2- or eosinophil-independent airway inflammation and BHR. Here, we describe the contributions of various functional T cell subsets to the development of bronchial asthma and propose a putative novel target for developing anti-asthma drugs that are effective in a wide range of asthma patients.

## 2. Th2 Cell-Mediated Eosinophilic Asthma

Clinical observations from the 1990s demonstrated that Th2 cytokines predominantly expressed in T cells accumulate in allergic tissues, whilst Mossman et al. proposed the Th1/Th2 theory [6,7,8]. It has since been shown that Th2 cells play a central role in the pathogenesis of asthma by secreting typical Th2 cytokines, such as interleukin (IL)-4, IL-5, and IL-13 [9,10,11].

The cytokine IL-4 plays multiple roles in allergic reactions and inflammation. Initially, IL-4 was found to promote antibody class switching from IgG to IgE in B cells [12]. The cross-linkage of IgE bound to FcεRI by specific allergens leads to the activation of mast cells and results in the secretion of several mediators such as histamine, proteases, and cytokines, which cause allergic symptoms and inflammatory responses [13]. IL-4 directly enhances the proliferative and mediator-secreting activity of mast cells [14] and has been shown to promote the differentiation of naïve T cells to Th2 cells [15,16,17]. Consistently, reduced peribronchial inflammation and eosinophil accumulation have been observed in the bronchial alveolar lavage (BALF) of allergen-immunized and allergen-challenged IL-4-deficient mice compared with wild type mice [18].

In addition to an IL-4-like IgE class-switching activity [19], IL-13 contributes to the effector phases and development of allergic inflammation via multiple bioactivities. IL-13 elicits goblet cell hyperplasia, mucus hyper production, and sub-epithelial airway fibrosis and promotes the expression of inducible nitric oxide (NO) synthase in airway epithelial cells, which increases fractional exhaled NO [20,21,22,23,24,25]. Using IL-13-deficient mice, IL-13 has been shown to have an essential role in BHR [23,26], for example, increasing the contraction and proliferation of airway smooth muscle cells was shown to be a possible mechanism of IL-13-mediated BHR in [27].

IL-5, initially identified as a T cell-derived B cell-activating factor [28], is strongly implicated in eosinophil-dependent inflammation and allergic responses. IL-5 regulates the terminal differentiation, maturation, proliferation, recruitment, and survival of eosinophils. Basic and clinical studies have revealed that IL-5 is essential for the development of BHR, at least in Th2 cell-mediated asthma, by reducing allergen-induced airway eosinophilic inflammation via its neutralizing antibody [29,30,31].

Consistent with the evidence demonstrating the crucial contribution of Th2 cells and cytokines to asthma, activated eosinophils have been found to play a major role in asthma pathogenesis, for example, a direct relationship between the degree of eosinophil accumulation and disease severity and exacerbation frequency was reported [32,33,34]. Since eosinophils release various cytotoxic proteins, such as major basic protein, eosinophil cationic protein, and cysteinyl leukotrienes (CysLTs) [35], their damage to the bronchial epithelium has been recognized as a possible primary mechanism underlying BHR in asthma patients. Surprisingly, the first animal model of bronchial asthma using eosinophil-deficient mice demonstrated that eosinophils were dispensable in the development of allergen-induced BHR [36]. Ourselves and Lee et al. obtained contradictory findings wherein allergen-induced BHR was significantly reduced in allergen-immunized or allergen-specific Th2 cell-transferred eosinophil-deficient mice [37,38]. Furthermore, Walsh et al. demonstrated that the dependency of murine models of BHR on eosinophils differed between mouse strains [39], suggesting that eosinophils participate differently in BHR development due to different forms of asthma pathogenesis.

## 3. Pharmacological Therapies for Th2 Cell-Mediated Asthma

The Global Initiative for Asthma (GINA) [40] currently recommends drug treatments for asthma patients using a stepwise approach from low- to high-dose inhaled corticosteroids (ICS) alone or in combination with other controllers such as long-acting β2-agonist (LABA), leukotriene receptor antagonists, and theophylline. Corticosteroids strongly down-regulate Th2 cell activity, which may be why ICS have become the gold standard drugs for asthma therapy. Decreased Th2 cytokine protein and mRNA levels as well as decreased numbers of Th2 cytokine-expressing cells have been observed in bronchial biopsies and peripheral blood mononuclear cells of asthma patients after treatment with oral and inhaled steroids [41,42,43,44]. Steroids inhibit allergen-induced Th2 cytokine production and T cell proliferation in vitro [45] and suppress allergen-induced airway eosinophil infiltration and BHR in murine models of asthma [46].

Elevated serum IgE levels in response to common environmental allergens are characteristic of Th2 cell-mediated asthma. Since IgE-mediated stimulation is required for mast cell activation and degranulation, an IgE-targeting therapy has been developed. Omalizumab, a humanized IgE-specific and non-anaphylactic IgG1 antibody, is currently used to treat patients with moderate-to-severe asthma, with clinical trials confirming that Omalizumab reduces circulating free IgE levels and asthma exacerbation rates [47,48,49,50].

Mast cells activated by IgE cross-linkage release various chemical mediators by degranulation, among which CysLTs such as LTC4, D4, and E4 are thought to play a role in asthma pathophysiology by inducing bronchoconstriction, mucus secretion, inflammatory cell recruitment and activation, fibrosis, and pulmonary edema formation. Furthermore, CysLTs are one of the most potent endogenous bronchoconstrictors, with inhaled CysLTs having broncho-constricting effects that are 100 to 1000 times stronger than histamine in normal subjects and asthma patients [51,52]. CysLTs are synthesized from arachidonic acid via the 5-lipozygenase pathway. In addition to mast cells, eosinophils produce large amounts of CysLTs [53]. Although there are two types of CysLT receptors, CysLT receptors 1 and 2, the major CysLTs bioactivity related with asthma phenotypes is mediated via CysLT receptor 1 [54,55,56]. Based on these findings and clinical trials indicating reduced asthma exacerbation and improved lung function in patients with mild-to-moderate asthma [57,58,59,60], 5-lipoxygenase inhibitors such as zileuton and CysLT receptor 1 antagonists such as montelukast have been approved.

Due to the successful development of anti-asthma drugs targeting cells and molecules in Th2 cell-related cascades, Th2 cytokines have become the next targets for treating asthma. The IL-5-producing capacity of CD4^+^ T cells is higher in both atopic and nonatopic patients with asthma [61]. Large numbers of T cells expressing IL-5 mRNA have been detected in the bronchial mucosa and sputum of asthmatic patients [62,63], whilst the BALF of atopic and nonatopic asthmatics exhibit increased IL-5 concentrations [64,65]. The IL-5-dependency of allergen-induced airway eosinophilic inflammation in murine models of asthma was confirmed using anti-IL-5 neutralizing antibodies [66,67]. Monoclonal antibodies targeting IL-5 (Mepolizumab) and IL-5 receptor α (Benralizumab) were recently approved to treat asthma; however, initial clinical studies on Mepolizumab have yielded conflicting findings. BHR was not alleviated in patients with atopic asthma even though peripheral eosinophils had almost completely disappeared [31,68]; conversely, in steroid-dependent and high sputum eosinophil patients, Mepolizumab significantly improved primary efficacy endpoints by reducing the number of severe exacerbations and oral glucocorticoid dose [69,70,71,72]. These findings clearly suggest that the pathogenesis of asthma displays at least two endotypes, IL-5-eosinophil cascade-dependent and cascade-independent.

Since IL-4 is a multifunctional cytokine that acts on various allergy-related cells, several attempts have been made to develop an anti-IL-4 therapy whilst the successful inhibition of airway inflammation in a murine model of asthma [73] has led to clinical trials of soluble IL-4 receptors (IL-4R) for treating asthma. Altrakincept is a recombinant soluble human IL-4R alpha subunit (IL4Rα), designated to antagonize the interaction between IL-4 and IL-4R expressed on the surface of target cells. Preliminary studies of Altrakincept in steroid-dependent atopic asthmatics demonstrated its potential to improve symptoms [74]; however, significant improvements were not observed in the later phase III trials [75]. Likewise, Pascolizumab, a humanized monoclonal antibody against IL-4 was shown to be ineffective for treating asthma [76].

Neutralizing IL-13, another Th2 cytokine, has shown promise for treating BHR and airway remodeling in animal models [77]; however, the usefulness of anti-IL-13 antibodies in clinical studies for asthma patients is controversial. Corren et al. performed a randomized, double-blind, placebo-controlled phase II study on the anti-IL13 monoclonal antibody (Lebrikizumab) [78]. Treatment with Lebrikizumab for 12 weeks significantly improved the forced expiratory volume in 1 second (FEV1) of 219 adult patients with asthma inadequately controlled by ICS therapy. Interestingly, patients with higher serum periostin levels exhibited greater improvements in lung function following Lebrikizumab treatment. However, Noonan et al. reported no significant difference between the FEV1 of mild asthmatics in Lebrikizumab- and placebo-treated groups in a randomized, double-blind, placebo-controlled phase II study [79]. Other phase II studies of Lebrikizumab in uncontrolled asthma patients have also reported discrepant results [80,81]. Although Lebrikizumab was found to significantly reduce asthma exacerbations in the primary analysis populations of phase III studies [82,83], it has not yet been approved for the treatment of asthma.

There is an obvious reason for the weak efficacy of asthma therapies targeting IL-4 and IL-13 since both cytokines share the same signaling pathway [84]. IL-4 exhibits its bioactivity by binding with either the type I IL-4R (heterodimer of IL-4Rα and the common γ chain) or the type II IL-4R (heterodimer of IL-4Rα and IL-13 receptor α1 chain). In addition to binding with type II IL-4R, IL-13 also associates with the IL-13 receptor α2 chain. Dupilumab is a humanized monoclonal antibody against IL-4R that blocks both IL-4- and IL-13-mediated signaling via type I and II IL-4R. Therefore, the expected effects of Dupilumab differ from those of solubilized IL-4Rα, anti-IL-4, and anti-IL-13. Wenzel et al. reported the initial results of a Dupilumab clinical trial in 2013 [85]. Patients with persistent, moderate to severe asthma, elevated blood and sputum eosinophils, and using medium- to high-dose to ICS plus LABAs exhibited significant improvements in lung function and asthma exacerbation rates following Dupilumab treatment, whilst serum levels of biomarkers associated with Th2-driven inflammation were reduced [85]. In other phase II and III studies, Dupilumab-treated patients with uncontrolled asthma displayed significant and sustainable improvements in lung function with greater benefits observed in patients with higher levels of Th2 markers, as evidenced by eosinophils and exhaled NO levels [86,87,88]. In addition to being approved for treating patients with atopic dermatitis in March 2017, the FDA approved Dupilumab as an add-on maintenance therapy for moderate-to-severe asthma in October 2018 [89].

## 4. Th1 and Th17 Cell-Mediated Neutrophilic Asthma

Various types of drugs targeting the Th2-related cascade have been approved for treating asthma, clearly suggesting that most asthma patients can be classified as the Th2-favored endotype. However, almost all Th2-related drugs other than steroids can only be used on certain populations of asthma patients, suggesting that there are a substantial number of patients whose pathophysiological condition is not mainly mediated by the Th2 cell-initiated cascade. These non-Th2 endotype patients appear to consist of multiple subgroups and often do not respond to inhaled corticosteroids (ICS) [90].

Consistent with classical Th1/Th2 theory, administering Th1-inducing factors, such as IL-12, bacterial components, and oligonucleotides inhibits allergen-induced murine airway inflammation [91,92,93]. However, the first report demonstrating the direct role of Th1 cells in a mouse model of asthma revealed that the adoptive transfer of allergen-specific Th1 cells failed to suppress Th2 cell-mediated BHR and caused severe inflammatory responses [94]. Moreover, Th1 cells have the potential to cause BHR accompanied by the massive airway accumulation of neutrophils, but not eosinophils [94]. Li et al. reported that IL-27 produced by pulmonary macrophage in response to combined stimulation with IFN-γ plus lipopolysaccharide and IFN-γ cooperatively induce steroid-resistant BHR through MyD88-dependent mechanisms [95]. In a clinical trial of patients with mild asthma, recombinant human IL-12 reduced the numbers of blood and sputum eosinophils but did not significantly affect BHR or late asthmatic responses [96].

In 2005, Th17 cells were identified as a new lineage of helper T cells that secrete several cytokines including IL17A, IL-17F, and IL-22 [97,98]. In mouse models, not only Th2 and Th1 cells but also Th17 cells have been shown to induce BHR. Similar to Th1 cell-mediated responses, Th17 cell-mediated airway inflammation is accompanied by massive neutrophil accumulation without substantial eosinophil migration [46]. Although the accumulation of neutrophils by Th1 cells in the lungs was reported to be caused by CXC chemokines [99], the mechanisms underlying Th17 cell-mediated BHR and airway neutrophilia are mostly unclear. Treatment with Dexamethasone (Dex) did not affect allergen-induced BHR but exacerbated airway neutrophilia in Th17 cell-transferred mice [46], whilst Dex suppressed eosinophil accumulation in Th2 cell-transferred mice [22]. Consistently, corticosteroids have been shown to suppress neutrophil apoptosis [100,101]. Several human studies have also suggested that Th17 cytokine levels and the extent of airway neutrophilia correlate with disease severity in asthma patients [102]. IL-17A acts on a wide range of cells, including epithelial and fibroblast cells, via IL17 receptor A (IL17RA) [103,104]. IL17A also regulates the chemokine release, proliferation, and contraction of airway smooth muscle cells [105,106,107,108], suggesting that IL17A plays a substantial role in the development of lung inflammation via multiple processes. Therefore, blocking the IL-17-mediated signaling cascade has been developed as a strategy for treating asthma. The efficacy of Brodalumab, a humanized monoclonal antibody that binds to IL17RA, was evaluated in patients with inadequately controlled moderate-to-severe asthma in a randomized controlled study in 2013 [109]. Although Brodalumab had no significant beneficial effect in asthma patients, it was recently approved to treat plaque psoriasis, another inflammatory disorder [110].

## 5. Th9 Cells

**Figure 1:**
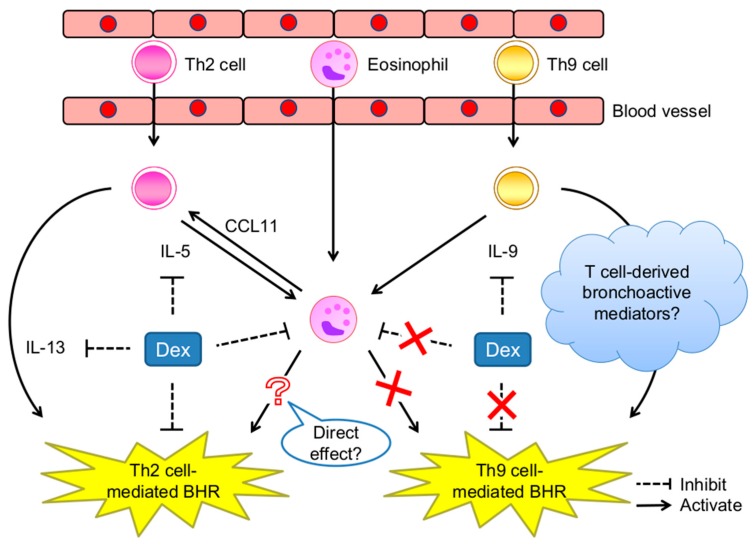
Schematic mechanisms of eosinophil-independent and steroid-resistant bronchial hyperresponsiveness (BHR) mediated by Th9 cells compared with Th2-mediated responses.

IL-9, a pleiotropic cytokine, induces mast cell proliferation, goblet cell hyperplasia, IL-13 production, eosinophil influx and local maturation, and BHR [97,98,99,100] (Figure 1). Classically, IL-9 has been considered a Th2 cytokine and has been implicated in allergic asthma and parasitic infections [111]. In 2008, a Th cell subset was identified to preferentially produce IL9 and these cells were named Th9 cells [112,113]. Th9 cells differentiate from naïve T cells in the presence of IL-4 and transforming growth factor-β (TGF-β) [112,113]. Although their transcriptional program has not been fully elucidated, several characteristic transcription factors, including STAT6, GATA3, PU.1, and IRF4, have been shown to participate in Th9 cell polarization [113,114,115,116,117,118]. IL-4 activates the STAT6 signaling pathway which induces GATA3, a master regulator of Th2 cells, and IRF4. GATA3 expression has been shown to increase IL-9 production in Th9 cells in several, but not all, studies [112,115,116]. GATA3 may negatively regulate the forkhead family transcription factor (Foxp3), which is a master regulator of regulatory T (Treg) cells [119]. Interestingly, the development of both Treg and Th9 cells requires TGF-β, whose signaling induces PU.1 expression which enhances IL-9 production during Th9 development and interferes with GATA3 function. Furthermore, TGF-β induces the expression of IRF4; STAT6 and IRF4 have been shown to directly bind to the *Il9* promoter. Although IRF4, STAT6, and GATA3 play important roles in the development of several other Th cells, the combinations of cytokine signals activating this transcription factor network are crucial for Th9 cell differentiation.

Th9 cells are associated with various diseases such as autoimmunity and other pathogen-mediated immunomodulatory disorders [120,121,122,123,124], whilst several studies have reported that Th9 cells have a critical role in anti-tumor immunity [122,123,125,126]. Purwar et al. showed that Th9 cells have a greater anti-tumor effect than other effector T cells, such as Th1 and Th17 cells in mouse models with adoptive transfer [122]. Th9 cells promote the activation of adaptive anti-tumor immune responses via IL-9 secretion, which activates mast cells that exhibit tumor growth-preventing activities [122]. In addition, Li et al. reported that Th9 cells elicit strong host anti-tumor CD8^+^ cytotoxic lymphocyte (CTL) responses by promoting the CCL20/CCL6-dependent recruitment of dendritic cells (DCs) to tumor tissues [123]. Although the ability of Th9 cells to directly trigger cancer cell death remains unclear, Purwar et al. showed that Th9 cells derived from OT-II transgenic mice effectively killed OVA-expressing tumor cells [122] and noted that Th9 cells expressed high levels of granzyme B. Down-regulating granzyme B in Th9 cells reduced their anti-tumor effects against melanoma cells.

## 6. Th9 Cells Induce BHR Accompanied by but Not Dependent on Eosinophil Infiltration

**Figure 2:**
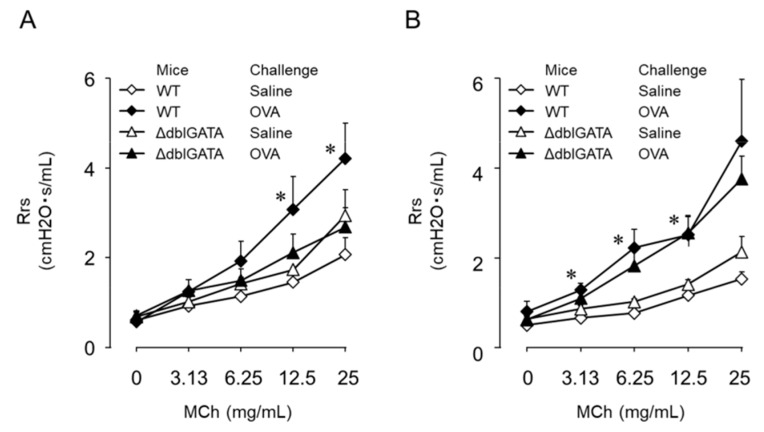
Antigen-induced airway inflammation in Th2 and Th9 cell-transferred mice. Th2 (**A**) or Th9 (**B**) cell-transferred wild-type (WT) and eosinophil-deficient ΔdblGATA mice were challenged with ovalbumin (OVA) or saline. Seventy-two hours after the challenge, bronchial responsiveness to inhaled methacholine (MCh) was assessed. Data are expressed as mean ± SEM of 3–10 animals. * *p* < 0.05 compared with saline-challenged WT mice. (Reference [38], modified).

Th9 cells have been shown to have substantial roles in allergic diseases in addition to their anti-tumor effects. Like Th2 cells, Th9 cells have the potential to induce airway eosinophilic inflammation accompanied by BHR [114,127]; however, the mechanisms of Th9 cell-mediated BHR are complicated. We examined the contribution of Th9 cells to asthma pathogenesis using an original mouse model. As observed in mice transferred with in vitro-differentiated allergen-specific Th2 cells, BHR and eosinophil infiltration were induced in Th9 cell-transferred mice upon allergen challenge [39,127] (Figure 1 and Figure 2). Xiao et al. reported that OX40 signaling in T cells induced Th9 cells and airway inflammation [128], whilst Kerzerho et al. reported that chronic exposure to *Aspergillus fumigatus* increased Th9 cell development in mouse lungs [129]. Clinically, the peripheral blood of patients with allergic asthma has been found to contain higher Th9 cell and IL-9 concentrations than healthy subjects [130,131]. Therefore, a randomized, placebo-control, double-blind, multicenter, parallel-group study on an anti-IL-9 monoclonal Ab in patients with uncontrolled moderate-to-severe asthma was performed in 2013; however, no significant improvements in predicted FEV1 % were obtained [132].

Our mouse study supported these clinical results, demonstrating that Th9 cell-mediated BHR was not affected by IL-9 neutralization. The dispensable nature of IL-10, another Th9-derived cytokine, was confirmed in mice transferred treated with IL-10-deficient Th9 cells. Furthermore, Th9-mediated BHR was substantially increased in eosinophil-deficient mice in contrast to the significant eosinophil-dependency observed in Th2 cell-mediated BHR [39,127] (Figure 1 and Figure 2). Our results contradict the report of Staudt et al., which demonstrated that BHR was downregulated by anti-IL-9 antibodies [114]. The reason for the discrepancy is unclear; however, they used RAG-2^−/−^ mice as Th9 cell recipients and challenged with the allergen for 6 consecutive days. Although chronic allergen exposure might increase IL-9 dependency in BHR development, the ineffectiveness of IL-9 neutralization therapy for asthma patients suggests that essential mediators of asthma pathogenesis other than IL-9 are produced by Th9 cells.

**Figure 3:**
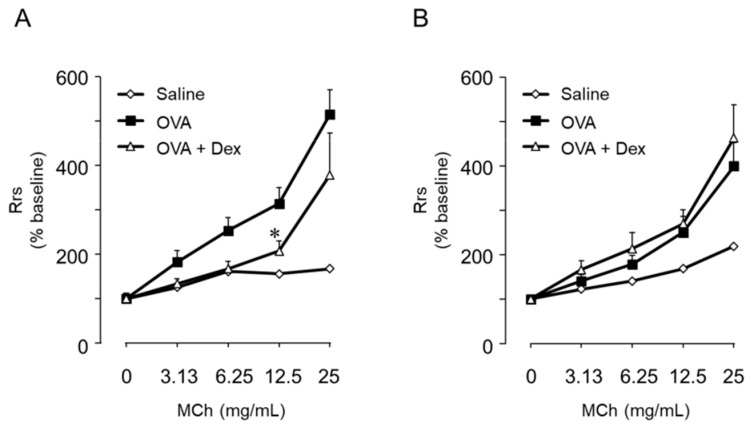
Dexamethsone (Dex) did not attenuate Th9 cell-mediated airway inflammation. Th2 (**A**) or Th9 (**B**) cell-transferred mice were challenged with ovalbumin (OVA) or saline. Mice were treated with either Dex (5 mg/kg) or phosphate buffered saline (PBS) as a control twice, at 1 h before and at 24 h after the OVA challenge, by subcutaneous injection. Seventy-two hours after the challenge, the bronchial responsiveness to inhaled MCh was assessed. Data are expressed as mean ± SEM of 5–7 animals. * *p* < 0.05, compared with OVA-challenged mice. (Reference [133], modified).

Th2- and Th9-mediated BHR also exhibit different responses to steroids. Consistent with the level of expression of glucocorticoid receptors in Th2 and Th9 cells, the typical cytokine production of these cells was similarly suppressed by Dex. However, allergen-induced lung eosinophilia and BHR were suppressed by Dex in mice transferred with Th2 cells but not Th9 cells [133] (Figure 1 and Figure 3). Although it has been suggested that IL-9 is involved in steroid-resistant asthma [132], the reason underlying this difference remains unclear. By monitoring the dynamics of allergen-specific T cells, we confirmed that Dex suppressed the allergen-induced migration of Th2, not Th9 cells [38]. These observations suggest innovative mechanisms by which steroids have a strong efficacy on allergic inflammation; reductions in Th2 cells but not eosinophils in the lungs may be a primary mechanism underlying the Dex-induced suppression of BHR in Th2 cell-mediated BHR.

T cells and T cell cytokines, particularly IL-5, are essential for the accumulation of eosinophils in the lungs, as described above; however, it has been suggested that eosinophils have a crucial role in T cell migration. Although mouse eosinophils are characterized by CCR3 and Siglec-F expression and the absence of CD11c, several eosinophil subpopulations have recently been identified. Siglec-F^+^Gr1^hi^ eosinophils accumulate in the lungs of allergen-challenged mice and maintain T cell-active cytokines [134]. The adoptive transfer of eosinophils and delivery of CCL11 into the lungs of eosinophil-deficient mice induced BHR development and T cell infiltration [39] (Figure 1). Consistently, Walsh et al. demonstrated that accumulated T cells contribute towards the development of eosinophil-dependent BHR [39]. The differential contribution of eosinophils and differences in the steroid-responsiveness of Th2- and Th9-mediated BHR might be caused by differences in the chemotactic activity of individual Th subsets.

Although the specific contributions of eosinophils to Th2 cell-mediated BHR have yet to be elucidated, previous reports have demonstrated eosinophil-independent BHR in mice transferred with multiple Th subsets (Th1, Th9, and Th17 cells) and suggested the existence of unknown BHR-inducing mediator(s) that are commonly produced by various Th cells. T cells are capable of producing bronchoactive mediators, including acetylcholine [135]. Moreover, we identified a higher-molecular-weight substance produced by murine T cells that induces the contraction of bronchial smooth muscle cells [136]. Allergen-induced late-phase airway obstruction was observed in mice transferred with OVA-reactive T cell clones [137]. T cell-derived bronchoactive mediators may contribute to BHR development by augmenting the basal tonus of bronchial smooth muscle.

Bronchial smooth muscle contraction is initiated by an increase in the intracellular Ca^2+^ concentration, which sequentially activates Ca^2+^-calmodulin and myosin light chain kinase (MLCK). MLCK phosphorylates the myosin light chain and promotes the formation of cross-bridges between myosin and actin, which generates the sliding force to contract smooth muscle (Ca^2+^ dynamics). The myosin light chain (MLC) is dephosphorylated and inactivated by MLC phosphatase (MLCP), which induces smooth muscle relaxation. Therefore, the balance between MLCK and MLCP plays a role in regulating smooth muscle contractile responses [138,139]. MLCP consists of three subunits: a protein phosphatase-1 catalytic subunit δ (also named beta), a myosin-targeting subunit1 (MYPT1), and an accessory 20 kDa subunit (M20). Recent studies have shown that several T cell cytokines such as IL-13, IL17A, and TNF-α increase the levels of the small GTPase, RhoA, and its effector Rho-kinase, ROCK2, via NF-κB. ROCK2 phosphorylates MYPT1 which suppresses MLCP activity; thus, these cytokines can enhance bronchial smooth muscle Ca^2+^ sensitivity and contraction [27,108,140,141]. Furthermore, we recently elucidated that protein kinase C (PKC)-potentiated phosphatase inhibitor protein of 17 kDa (CPI-17) is involved in down-regulating MLCP and regulates the activity of smooth muscle cells and T cells (unpublished data).

With respect to hyperresponsiveness and commonality in Th subsets, we recently showed that allergen-induced nasal hyperresponsiveness (NHR), evaluated by increasing non-specific stimuli-evoked sneezing responses, was induced in mice transferred with not only Th2 cells but also Th1 and Th17 cells [142]. In contrast to eosinophil dependency in Th2 cell-mediated BHR, eosinophils were found to be dispensable in NHR even in Th2 cell-transferred mice. In immunized and Th2-transferred mice, allergen-induced NHR was suppressed by Dex treatment, although its effect on the other Th subset-mediated responses has not yet been examined. Focusing on the tissue-specific physiological reactions of bronchial smooth muscle contraction and sneezing may be useful for identifying hyperresponsiveness-inducing factors in various Th subsets.

## 7. Conclusions

Although Th2 cell-mediated eosinophilic inflammation is an important disease mechanism in the majority of patients with bronchial asthma, a substantial population of patients exhibit non-Th2 endotypes. We suggest novel, unknown BHR-inducing mechanisms in which eosinophils are dispensable but various Th subsets are involved. Since the restricted usage of Th2-targeted drugs in different patient types indicates unmet therapeutic needs, future drugs targeting Th subset-derived BHR-inducing factors promise to be effective for a wide range of patients with different asthma pathogenesis.

## References

[1] GBD 2015 Disease and Injury Incidence and Prevalence Collaborators (2016). Global, regional, and national incidence, prevalence, and years lived with disability for 310 diseases and injuries, 1990–2015: A systematic analysis for the Global Burden of Disease Study 2015. Lancet.

[2] Wenzel S.E. (2006). Asthma: Defining of the persistent adult phenotypes. Lancet.

[3] Lötvall J., Akdis C.A., Bacharier L.B., Bjermer L., Casale T.B., Custovic A., Lemanske R.F., Wardlaw A.J., Wenzel S.E., Greenberger P.A. (2011). Asthma endotypes: A new approach to classification of disease entities within the asthma syndrome. J. Allergy Clin. Immunol..

[4] Haldar P., Pavord I.D., Shaw D.E., Berry M.A., Thomas M., Brightling C.E., Wardlaw A.J., Green R.H. (2008). Cluster analysis and clinical asthma phenotypes. Am. J. Respir. Crit. Care Med..

[5] Akdis C.A., Bachert C., Cingi C., Dykewicz M.S., Hellings P.W., Naclerio R.M., Schleimer R.P., Ledford D. (2013). Endotypes and phenotypes of chronic rhinosinusitis: A PRACTALL document of the European Academy of Allergy and Clinical Immunology and the American Academy of Allergy, Asthma & Immunology. J. Allergy Clin. Immunol..

[6] Mosmann T.R., Cherwinski H., Bond M.W., Giedlin M.A., Coffman R.L. (1986). Two types of murine helper T cell clone. I. Definition according to profiles of lymphokine activities and secreted proteins. J. Immunol..

[7] Robinson D.S., Hamid Q., Ying S., Tsicopoulos A., Barkans J., Bentley A.M., Corrigan C., Durham S.R., Kay A.B. (1992). Predominant TH2-like bronchoalveolar T-lymphocyte population in atopic asthma. N. Engl. J. Med..

[8] Van Reijsen F.C., Bruijnzeel-Koomen C.A., Kalthoff F.S., Maggi E., Romagnani S., Westland J.K., Mudde G.C. (1992). Skin-derived aeroallergen-specific T-cell clones of Th2 phenotype in patients with atopic dermatitis. J. Allergy Clin. Immunol..

[9] Wills-Karp M. (1999). Immunological basis of antigen-induced airways hyperresponsiveness. Annu. Rev. Immunol..

[10] Kay A.B. (1991). Asthma and inflammation. J. Allergy Clin. Immunol..

[11] Bochner B.S., Undem B.J., Lichtenstein L.M. (1994). Immunological aspects of allergic asthma. Annu. Rev. Immunol..

[12] Snapper C.M., Finkelman F.D., Paul W.E. (1988). Differential regulation of IgG1 and IgE synthesis by interleukin 4. J. Exp. Med..

[13] Metcalfe D.D., Baram D., Mekori Y.A. (1997). Mast cells. Physiol. Rev..

[14] Bischoff S.C., Sellge G., Lorentz A., Sebald W., Raab R., Manns M.P. (1999). IL-4 enhances proliferation and mediator release in mature human mast cells. Proc. Natl. Acad. Sci. USA.

[15] Seder R.A., Paul W.E., Davis M.M., Fazekas de St Groth B. (1992). The presence of interleukin 4 during in vitro priming determines the lymphokine-producing potential of CD4^+^ T cells from T cell receptor transgenic mice. J. Exp. Med..

[16] Hsieh C.S., Heimberger A.B., Gold J.S., O’Garra A.K., Murphy M. (1992). Differential regulation of T helper phenotype development by interleukins 4 and 10 in an αβ T-cell-receptor transgenic system. Proc. Natl. Acad. Sci. USA.

[17] Swain S.L., Weinberg A.D., English M., Huston G. (1990). IL-4 directs the development of Th2-like helper effectors. J. Immunol..

[18] Brusselle G.G., Kips J.C., Tavernier J.H., van der Heyden J.G., Cuvelier C.A., Pauwels R.A., Bluethmann H. (1994). Attenuation of allergic airway inflammation in IL-4 deficient mice. Clin. Exp. Allergy..

[19] Emson C.L., Bell S.E., Jones A., Wisden W., McKenzie A.N. (1998). Interleukin (IL)-4-independent induction of immunoglobulin (Ig)E, and perturbation of T cell development in transgenic mice expressing IL-13. J. Exp. Med..

[20] Wills-Karp M., Luyimbazi J., Xu X., Schofield B., Neben T.Y., Karp C.L., Donaldson D.D. (1998). Interleukin-13: Central mediator of allergic asthma. Science.

[21] Grünig G., Warnock M., Wakil A.E., Venkayya R., Brombacher F., Rennick D.M., Sheppard D., Mohrs M., Donaldson D.D., Locksley R.M. (1998). Requirement for IL-13 independently of IL-4 in experimental asthma. Science.

[22] Zhu Z., Homer R.J., Wang Z., Chen Q., Geba G.P., Wang J., Zhang Y., Elias J.A. (1999). Pulmonary expression of interleukin-13 causes inflammation, mucus hypersecretion, subepithelial fibrosis, physiologic abnormalities, and eotaxin production. J. Clin. Investig..

[23] Webb D.C., McKenzie A.N., Koskinen A.M., Yang M., Mattes J., Foster P.S. (2000). Integrated signals between IL-13, IL-4, and IL-5 regulate airways hyperreactivity. J. Immunol..

[24] Suresh V., Mih J.D., George S.C. (2007). Measurement of IL-13–Induced iNOS-Derived Gas Phase Nitric Oxide in Human Bronchial Epithelial Cells. Am. J. Respir. Cell Mol. Biol..

[25] Chibana K., Trudeau J.B., Mustovich A.T., Hu H., Zhao J., Balzar S., Chu H.W., Wenzel S.E. (2008). IL-13 induced increases in nitrite levels are primarily driven by increases in inducible nitric oxide synthase as compared with effects on arginases in human primary bronchial epithelial cells. Clin. Exp. Allergy.

[26] Walter D.M., McIntire J.J., Berry G., McKenzie A.N., Donaldson D.D., DeKruyff R.H., Umetsu D.T. (2001). Critical role for IL-13 in the development of allergen-induced airway hyperreactivity. J. Immunol..

[27] Chiba Y., Nakazawa S., Todoroki M., Shinozaki K., Sakai H., Misawa M. (2009). Interleukin-13 augments bronchial smooth muscle contractility with an up-regulation of RhoA protein. Am. J. Respir. Cell Mol. Biol..

[28] Kinashi T., Harada N., Severinson E., Tanabe T., Sideras P., Konishi M., Azuma C., Tominaga A., Bergstedt-Lindqvist S., Takahashi M. (1986). Cloning of complementary DNA encoding T-cell replacing factor and identity with B-cell growth factor II. Nature.

[29] Hamelmann E., Cieslewicz G., Schwarze J., Ishizuka T., Joetham A., Heusser C., Gelfand E.W. (1999). Anti-IL-5 but not anti-IgE prevents airway inflammation and airway hyperresponsiveness. Am. J. Respir. Crit. Care Med..

[30] Kumar R.K., Herbert C., Webb D.C., Li L., Foster P.S. (2004). Effects of anticytokine therapy in a mouse model of chronic asthma. Am. J. Respir. Crit. Care Med..

[31] Leckie M.J., ten Brinke A., Khan J., Diamant Z., O’Connor B.J., Walls C.M., Mathur A.K., Cowley H.C., Chung K.F., Djukanovic R. (2000). Effects of an interleukin-5 blocking monoclonal antibody on eosinophils, airway hyper-responsiveness, and the late asthmatic response. Lancet.

[32] Bousquet J., Chanez P., Lacoste J.Y., Barneon G., Ghavanian N., Enander I., Venge P., Ahlstedt S., Simony-Lafontaine J., Godard P. (1990). Eosinophilic inflammation in asthma. N. Engl. J. Med..

[33] Garcia G., Taille C., Laveneziana P., Bourdin A., Chanez P., Humbert M. (2013). Anti-interleukin-5 therapy in severe asthma. Eur. Respir. Rev..

[34] Price D.B., Rigazio A., Campbell J.D., Bleecker E.R., Corrigan C.J., Thomas M., Wenzel S.E., Wilson A.M., Small M.B., Gopalan G. (2015). Blood eosinophil count and prospective annual asthma disease burden: A UK cohort study. Lancet Respir. Med..

[35] McBrien C.N., Menzies-Gow A. (2017). The Biology of Eosinophils and Their Role in Asthma. Front. Med..

[36] Humbles A.A., Lloyd C.M., McMillan S.J., Friend D.S., Xanthou G., McKenna E.E., Ghiran S., Gerard N.P., Yu C., Orkin S.H. (2004). A critical role for eosinophils in allergic airways remodeling. Science.

[37] Lee J.J., Dimina D., Macias M.P., Ochkur S.I., McGarry M.P., O’Neill K.R., Protheroe C., Pero R., Nguyen T., Cormier S.A. (2004). Defining a link with asthma in mice congenitally deficient in eosinophils. Science.

[38] Saeki M., Kaminuma O., Nishimura T., Kitamura N., Mori A., Hiroi T. (2016). Th9 cells elicit eosinophil-independent bronchial hyperresponsiveness in mice. Allergol. Int..

[39] Walsh E.R., Sahu N., Kearley J., Benjamin E., Kang B.H., Humbles A., August A. (2008). Strain-specific requirement for eosinophils in the recruitment of T cells to the lung during the development of allergic asthma. J. Exp. Med..

[40] 2018 GINA Report, Global Strategy for Asthma Management and Prevention. https://ginasthma.org/wp-content/uploads/2018/04/wms-GINA-2018-report-V1.3-002.pdf.

[41] Doi S., Gemou-Engesaeth V., Kay A.B., Corrigan C.J. (1994). Polymerase chain reaction quantification of cytokine messenger RNA expression in peripheral blood mononuclear cells of patients with acute exacerbations of asthma: Effect of glucocorticoid therapy. Clin. Exp. Allergy.

[42] Gemou-Engesaeth V., Bush A., Kay A.B., Hamid Q., Corrigan C.J. (1997). Inhaled Glucocorticoid Therapy of Childhood Asthma Is Associated with Reduced Peripheral Blood T Cell Activation and ‘Th2-Type’ Cytokine mRNA Expression. Pediatrics.

[43] Gemou-Engesaeth V., Fagerhol M.K., Toda M., Hamid Q., Halvorsen S., Groegaard J.B., Corrigan C.J. (2002). Expression of activation markers and cytokine mRNA by peripheral blood CD4 and CD8 T cells in atopic and nonatopic childhood asthma: Effect of inhaled glucocorticoid therapy. Pediatrics.

[44] Wallin A., Sandström T., Cioppa G.D., Holgate S., Wilson S. (2002). The effects of regular inhaled formoterol and budesonide on preformed Th-2 cytokines in mild asthmatics. Respir. Med..

[45] Barrat F.J., Cua D.J., Boonstra A., Richards D.F., Crain C., Savelkoul H.F., de Waal-Malefyt R., Coffman R.L., Hawrylowicz C.M., O’Garra A. (2002). In vitro generation of interleukin 10-producing regulatory CD4^+^ T cells is induced by immunosuppressive drugs and inhibited by T helper type 1 (Th1)- and Th2-inducing cytokines. J. Exp. Med..

[46] McKinley L., Alcorn J.F., Peterson A., Dupont R.B., Kapadia S., Logar A., Henry A., Irvin C.G., Piganelli J.D., Ray A. (2008). TH17 cells mediate steroid-resistant airway inflammation and airway hyperresponsiveness in mice. J. Immunol..

[47] Milgrom H., Berger W., Nayak A., Gupta N., Pollard S., McAlary M., Taylor A.F., Rohane P. (2001). Treatment of childhood asthma with anti-immunoglobulin E antibody (omalizumab). Pediatrics.

[48] Buh R., Solèr M., Matz J., Townley R., O’Brien J., Noga O., Champain K., Fox H., Thirlwell J., Della Cioppa G. (2002). Omalizumab provides long-term control in patients with moderate-to-severe allergic asthma. Eur. Respir. J..

[49] Bousquet J., Cabrera P., Berkman N., Buhl R., Holgate S., Wenzel S., Fox H., Hedgecock S., Blogg M., Cioppa G.D. (2005). The effect of treatment with omalizumab, an anti-IgE antibody, on asthma exacerbations and emergency medical visits in patients with severe persistent asthma. Allergy.

[50] Holgate S.T., Djukanovic R., Casale T., Bousquet J. (2005). Anti-immunoglobulin E treatment with omalizumab in allergic diseases: An update on anti-inflammatory activity and clinical efficacy. Clin. Exp. Allergy.

[51] Dahlén S.E., Hedqvist P., Hammarström S., Samuelsson B. (1980). Leukotrienes are potent constrictors of human bronchi. Nature.

[52] Dahlén S.E., Hansson G., Hedqvist P., Björck T., Granström E., Dahlén B. (1983). Allergen challenge of lung tissue from asthmatics elicits bronchial contraction that correlates with the release of leukotrienes C4, D4, and E4. Proc. Natl. Acad. Sci. USA.

[53] Peters-Golden M., Henderson W.R. (2007). Leukotrienes. N. Engl. J. Med..

[54] Hay D.W., Muccitelli R.M., Tucker S.S., Vickery-Clark L.M., Wilson K.A., Gleason J.G., Hall R.F., Wasserman M.A., Torphy T.J. (1987). Pharmacologic profile of SK&F 104353: A novel, potent and selective peptidoleukotriene receptor antagonist in guinea pig and human airways. J. Pharmacol. Exp. Ther..

[55] Watanabe-Kohno S., Yasui K., Nabe T., Yamamura H., Horiba M., Ohata K. (1992). Significant role of peptide leukotrienes (p-LTs) in the antigen-induced contractions of human and guinea pig lung parenchymas and bronchi or tracheas in vitro. Jpn. J. Pharmacol..

[56] Yamaguchi T., Kohrogi H., Honda I., Kawano O., Sugimoto M., Araki S., Ando M.A. (1992). Novel leukotriene antagonist, ONO-1078, inhibits and reverses human bronchial contraction induced by leukotrienes C4 and D4 and antigen in vitro. Am. Rev. Respir. Dis..

[57] Knorr B., Matz J., Bernstein J.A., Nguyen H., Seidenberg B.C., Reiss T.F., Becker A. (1998). Montelukast for chronic asthma in 6- to 14-year-old children: A randomized, double-blind trial. Pediatric Montelukast Study Group. JAMA.

[58] Reiss T.F., Chervinsky P., Dockhorn R.J., Shingo S., Seidenberg B., Edwards T.B. (1998). Montelukast, a once-daily leukotriene receptor antagonist, in the treatment of chronic asthma: A multicenter, randomized, double-blind trial. Montelukast Clinical Research Study Group. Arch. Intern. Med..

[59] Israel E., Cohn J., Dube L., Drazen J.M. (1996). Effect of treatment with zileuton, a 5-lipoxygenase inhibitor, in patients with asthma. A randomized controlled trial. Zileuton Clinical Trial Group. JAMA.

[60] Nelson H., Kemp J., Berger W., Corren J., Casale T., Dube L., Walton-Bowen K., LaVallee N., Stepanians M. (2007). Efficacy of zileuton controlled-release tablets administered twice daily in the treatment of moderate persistent asthma: A 3-month randomized controlled study. Ann. Allergy Asthma Immunol..

[61] Shiota Y., Arikita H., Horita N., Hiyama J., Ono T., Yamakido M. (2002). Intracellular IL-5 and T-lymphocyte subsets in atopic and nonatopic bronchial asthma. J. Allergy Clin. Immunol..

[62] Hamid Q., Azzawi M., Ying S., Moqbel R., Wardlaw A.J., Corrigan C.J., Bradley B., Durham S.R., Collins J.V., Jeffery P.K. (1991). Expression of mRNA for interleukin-5 in mucosal bronchial biopsies from asthma. J. Clin. Investig..

[63] Truyen E., Coteur L., Dilissen E., Overbergh L., Dupont L.J., Ceuppens J.L., Bullens D.M. (2006). Evaluation of airway inflammation by quantitative Th1/Th2 cytokine mRNA measurement in sputum of asthma patients. Thorax.

[64] Walker C., Bode E., Boer L., Hansel T.T., Blaser K., Virchow J.C. (1992). Allergic and nonallergic asthmatics have distinct patterns of T-cell activation and cytokine production in peripheral blood and bronchoalveolar lavage. Am. Rev. Respir. Dis..

[65] Walker C., Bauer W., Braun R.K., Menz G., Braun P., Schwarz F., Hansel T.T., Villiger B. (1994). Activated T cells and cytokines in bronchoalveolar lavages from patients with various lung diseases associated with eosinophilia. Am. J. Respir. Crit. Care Med..

[66] Nagai H., Yamaguchi S., Inagaki N., Tsuruoka N., Hitoshi Y., Takatsu K. (1993). Effect of anti-IL-5 monoclonal antibody on allergic bronchial eosinophilia and airway hyperresponsiveness in mice. Life Sci..

[67] Kung T.T., Stelts D.M., Zurcher J.A., Adams G.K., Egan R.W., Kreutner W., Watnick A.S., Jones H., Chapman R.W. (1995). Involvement of IL-5 in a murine model of allergic pulmonary inflammation: Prophylactic and therapeutic effect of an anti-IL-5 antibody. Am. J. Respir. Cell Mol. Biol..

[68] Flood-Page P., Swenson C., Faiferman I., Matthews J., Williams M., Brannick L., Robinson D., Wenzel S., Busse W., Hansel T.T. (2007). A study to evaluate safety and efficacy of mepolizumab in patients with moderate persistent asthma. Am. J. Respir. Crit. Care Med..

[69] Ortega H., Chupp G., Bardin P., Bourdin A., Garcia G., Hartley B., Yancey S., Humbert M. (2014). The role of mepolizumab in atopic and nonatopic severe asthma with persistent eosinophilia. Eur. Respir. J..

[70] Nair P., Pizzichini M.M., Kjarsgaard M., Inman M.D., Efthimiadis A., Pizzichini E., Hargreave F.E., O’Byrne P.M. (2009). Mepolizumab for prednisone-dependent asthma with sputum eosinophilia. N. Engl. J. Med..

[71] Pavord I.D., Korn S., Howarth P., Bleecker E.R., Buhl R., Keene O.N., Ortega H., Chanez P. (2012). Mepolizumab for severe eosinophilic asthma (DREAM): A multicentre, double-blind, placebo-controlled trial. Lancet.

[72] Bel E.H., Wenzel S.E., Thompson P.J., Prazma C.M., Keene O.N., Yancey S.W., Ortega H.G., Pavord I.D., SIRIUS Investigators (2014). Oral glucocorticoid-sparing effect of mepolizumab in eosinophilic asthma. N. Engl. J. Med..

[73] Zhou C.Y., Crocker I.C., Koenig G., Romero F.A., Townley R.G. (1997). Anti-interleukin-4 inhibits immunoglobulin E production in a murine model of atopic asthma. J. Asthma.

[74] Borish L.C., Nelson H.S., Lanz M.J., Claussen L., Whitmore J.B., Agosti J.M., Garrison L. (1999). Interleukin-4 receptor in moderate atopic asthma. A phase I/II randomized, placebo-controlled trial. Am. J. Respir. Crit. Care Med..

[75] Akdis C.A. (2012). Therapies for allergic inflammation: Refining strategies to induce tolerance. Nat. Med..

[76] Hart T.K., Blackburn M.N., Brigham-Burke M., Dede K., Al-Mahdi N., Zia-Amirhosseini P., Cook R.M. (2002). Preclinical efficacy and safety of pascolizumab (SB 240683): A humanized anti-interleukin-4 antibody with therapeutic potential in asthma. Clin. Exp. Immunol..

[77] Tomlinson K.L., Davies G.C., Sutton D.J., Palframan R.T. (2010). Neutralisation of Interleukin-13 in Mice Prevents Airway Pathology Caused by Chronic Exposure to House Dust Mite. PLoS One.

[78] Corren J., Lemanske R.F., Hanania N.A., Korenblat P.E., Parsey M.V., Arron J.R., Harris J.M., Scheerens H., Wu L.C., Su Z. (2011). Lebrikizumab treatment in adults with asthma. N. Engl. J. Med..

[79] Noonan M., Korenblat P., Mosesova S., Scheerens H., Arron J.R., Zheng Y., Putnam W.S., Parsey M.V., Bohen S.P., Matthews J.G. (2013). Dose-ranging study of lebrikizumab in asthmatic patients not receiving inhaled steroids. J. Allergy Clin. Immunol..

[80] Scheerens H., Arron J.R., Zheng Y., Putnam W.S., Erickson R.W., Choy D.F., Harris J.M., Lee J., Jarjour N.N., Matthews J.G. (2014). The effects of lebrikizumab in patients with mild asthma following whole lung allergen challenge. Clin. Exp. Allergy.

[81] Hanania N.A., Noonan M., Corren J., Korenblat P., Zheng Y., Fischer S.K., Cheu M., Putnam W.S., Murray E., Scheerens H. (2015). Lebrikizumab in moderate-to-severe asthma: Pooled data from two randomised placebo-controlled studies. Thorax.

[82] U.S. National Institutes of Health LAVOLTA I Study Record. https://clinicaltrials.gov/ct2/show/NCT01868061.

[83] U.S. National Institutes of Health LAVOLTA II Study Record. https://clinicaltrials.gov/ct2/show/NCT01867125.

[84] Oh C.K., Geba G.P., Molfino N. (2010). Investigational therapeutics targeting the IL-4/IL-13/STAT-6 pathway for the treatment of asthma. Eur. Respir. Rev..

[85] Wenzel S., Ford L., Pearlman D., Spector S., Sher L., Skobieranda F., Wang L., Kirkesseli S., Rocklin R., Bock B. (2013). Dupilumab in persistent asthma with elevated eosinophil levels. N. Engl. J. Med..

[86] Thaçi D., Simpson E.L., Beck L.A., Bieber T., Blauvelt A., Papp K., Soong W., Worm M., Szepietowski J.C., Sofen H. (2016). Efficacy and safety of dupilumab in adults with moderate-to-severe atopic dermatitis inadequately controlled by topical treatments: A randomised, placebo-controlled, dose-ranging phase 2b trial. Lancet.

[87] Castro M., Corren J., Pavord I.D., Maspero J., Wenzel S., Rabe K.F., Busse W.W., Ford L., Sher L., FitzGerald J.M. (2018). Dupilumab efficacy and safety in moderateto-severe uncontrolled asthma. N. Engl. J. Med..

[88] Rabe K.F., Nair P., Brusselle G., Maspero J.F., Castro M., Sher L., Zhu H., Hamilton J.D., Swanson B.N., Khan A. (2018). Efficacy and Safety of Dupilumab in Glucocorticoid-Dependent Severe Asthma. N. Engl. J. Med..

[89] FDA approves asthma indication for Dupixent (dupilumab). http://www.news.sanofi.us/2018-10-19-FDA-approves-asthsma-indication-for-Dupixent-R-dupilumab.

[90] Wenzel S.E. (2012). Asthma phenotypes: The evolution from clinical to molecular approaches. Nat. Med..

[91] Gavett S.H., O’Hearn D.J., Li X., Huang S.K., Finkelman F.D., Wills-Karp M. (1995). Interleukin 12 inhibits antigen-induced airway hyperresponsiveness, inflammation, and Th2 cytokine expression in mice. J. Exp. Med..

[92] Kitagaki K., Jain V.V., Businga T.R., Hussain I., Kline J.N. (2002). Immunomodulatory effects of CpG oligodeoxynucleotides on established Th2 responses. Clin. Diagn. Lab. Immunol..

[93] Bortolatto J., Borducchi E., Rodriguez D., Keller A.C., Faquim-Mauro E., Bortoluci K.R., Mucida D., Gomes E., Christ A., Schnyder-Candrian S. (2008). Toll-like receptor 4 agonists adsorbed to aluminium hydroxide adjuvant attenuate ovalbumin-specific allergic airway disease: Role of MyD88 adaptor molecule and interleukin-12/interferon-gamma axis. Clin. Exp. Allergy.

[94] Hansen G., Berry G., DeKruyff R.H., Umetsu D.T. (1999). Allergen-specific Th1 cells fail to counterbalance Th2 cell–induced airway hyperreactivity but cause severe airway inflammation. J. Clin. Investig..

[95] Li J.J., Wang W., Baines K.J., Bowden N.A., Hansbro P.M., Gibson P.G., Kumar R.K., Foster P.S., Yang M. (2010). IL-27/IFN-γ induce MyD88-dependent steroid-resistant airway hyperresponsiveness by inhibiting glucocorticoid signaling in macrophages. J. Immunol..

[96] Bryan S.A., O’Connor B.J., Matti S., Leckie M.J., Kanabar V., Khan J., Warrington S.J., Renzetti L., Rames A., Bock J.A. (2000). Effects of recombinant human interleukin-12 on eosinophils, airway hyper-responsiveness, and the late asthmatic response. Lancet.

[97] Harrington L.E., Hatton R.D., Mangan P.R., Turner H., Murphy T.L., Murphy K.M., Weaver C.T. (2005). Interleukin 17-producing CD4+ effector T cells develop via a lineage distinct from the T helper type 1 and 2 lineages. Nat. Immunol..

[98] Liang S.C., Tan X.Y., Luxenberg D.P., Karim R., Dunussi-Joannopoulos K., Collins M., Fouser L.A. (2006). Interleukin (IL)-22 and IL-17 are coexpressed by Th17 cells and cooperatively enhance expression of antimicrobial peptides. J. Exp. Med..

[99] Takaoka A., Tanaka Y., Tsuji T., Jinushi T., Hoshino A., Asakura Y., Mita Y., Watanabe K., Nakaike S., Togashi Y. (2001). A critical role for mouse CXC chemokine(s) in pulmonary neutrophilia during Th type 1-dependent airway inflammation. J. Immunol..

[100] Liles W.C., Dale D.C., Klebanoff S.J. (1995). Glucocorticoids inhibit apoptosis of human neutrophils. Blood.

[101] Saffar A.S., Ashdown H., Gounni A.S. (2011). The molecular mechanisms of glucocorticoids-mediated neutrophil survival. Curr. Drug Targets.

[102] Chesné J., Braza F., Mahay G., Brouard S., Aronica M., Magnan A. (2014). IL-17 in severe asthma. Where do we stand?. Am. J. Respir. Crit. Care Med..

[103] Molet S., Hamid Q., Davoine F., Nutku E., Taha R., Page N., Olivenstein R., Elias J., Chakir J. (2001). IL-17 is increased in asthmatic airways and induces human bronchial fibroblasts to produce cytokines. J. Allergy Clin. Immunol..

[104] Kawaguchi M., Kokubu F., Kuga H., Matsukura S., Hoshino H., Ieki K., Imai T., Adachi M., Huang S.K. (2001). Modulation of bronchial epithelial cells by IL-17. J. Allergy Clin. Immunol..

[105] Rahman M.S., Yamasaki A., Yang J., Shan L., Halayko A.J., Gounni A.S. (2006). IL-17A induces eotaxin-1/CC chemokine ligand 11 expression in human airway smooth muscle cells: Role of MAPK (Erk1/2, JNK, and p38) pathways. J. Immunol..

[106] Al-Alwan L.A., Chang Y., Baglole C.J., Risse P.A., Halayko A.J., Martin J.G., Eidelman D.H., Hamid Q. (2012). Autocrine-regulated airway smooth muscle cell migration is dependent on IL-17-induced growth-related oncogenes. J. Allergy Clin. Immunol..

[107] Chang Y., Al-Alwan L., Risse P.A., Halayko A.J., Martin J.G., Baglole C.J., Eidelman D.H., Hamid Q. (2012). Th17-associated cytokines promote human airway smooth muscle cell proliferation. FASEB J..

[108] Kudo M., Melton A.C., Chen C., Engler M.B., Huang K.E., Ren X., Wang Y., Bernstein X., Li J.T., Atabai K. (2012). IL-17A produced by αβ T cells drives airway hyper-responsiveness in mice and enhances mouse and human airway smooth muscle contraction. Nat. Med..

[109] Busse W.W., Holgate S., Kerwin E., Chon Y., Feng J., Lin J., Lin S.L. (2013). Randomized, double-blind, placebo-controlled study of brodalumab, a human anti-IL-17 receptor monoclonal antibody, in moderate to severe asthma. Am. J. Respir. Crit. Care Med..

[110] FDA Approves New Psoriasis Drug. https://www.fda.gov/news-events/press-announcements/fda-approves-new-psoriasis-drug.

[111] Faulkner H., Humphreys N., Renauld J.C., Van Snick J., Grencis R. (1997). Interleukin-9 is involved in host protective immunity to intestinal nematode infection. Eur. J. Immunol..

[112] Dardalhon V., Awasthi A., Kwon H., Galileos G., Gao W., Sobel R.A., Mitsdoerffer M., Strom T.B., Elyaman W., Ho I.C. (2008). IL-4 inhibits TGF-beta-induced Foxp3+ T cells and, together with TGF-beta, generates IL-9+ IL-10+ Foxp3(-) effector T cells. Nat. Immunol..

[113] Veldhoen M., Uyttenhove C., van Snick J., Helmby H., Westendorf A., Buer J., Martin B., Wilhelm C., Stockinger B. (2008). Transforming growth factor-beta ‘reprograms’ the differentiation of T helper 2 cells and promotes an interleukin 9-producing subset. Nat. Immunol..

[114] Staudt V., Bothur E., Klein M., Lingnau K., Reuter S., Grebe N., Gerlitzki B., Hoffmann M., Ulges A., Taube C. (2010). Interferon-regulatory factor 4 is essential for the developmental program of T helper 9 cells. Immunity.

[115] Goswami R., Jabeen R., Yagi R., Pham D., Zhu J., Goenka S., Kaplan M.H. (2012). STAT6-dependent regulation of Th9 development. J. Immunol..

[116] Chang H.C., Sehra S., Goswami R., Yao W., Yu Q., Stritesky G.L., Jabeen R., McKinley C., Ahyi A.N., Han L. (2010). The transcription factor PU.1 is required for the development of IL-9-producing T cells and allergic inflammation. Nat. Immunol..

[117] Jabeen R., Goswami R., Awe O., Kulkarni A., Nguyen E.T., Attenasio A., Walsh D., Olson M.R., Kim M.H., Tepper R.S. (2013). Th9 cell development requires a BATF-regulated transcriptional network. J. Clin. Invest..

[118] Olson M.R., Verdan F.F., Hufford M.M., Dent A.L., Kaplan M.H. (2016). STAT3 Impairs STAT5 Activation in the Development of IL-9-Secreting T Cells. J. Immunol..

[119] Mantel P.Y., Kuipers H., Boyman O., Rhyner C., Ouaked N., Rückert B., Karagiannidis C., Lambrecht B.N., Hendriks R.W., Crameri R. (2007). GATA3-driven Th2 responses inhibit TGF-beta1-induced FOXP3 expression and the formation of regulatory T cells. PLoS Biol..

[120] Jäger A., Dardalhon V., Sobel R.A., Bettelli E., Kuchroo V.K. (2009). Th1, Th17, and Th9 effector cells induce experimental autoimmune encephalomyelitis with different pathological phenotypes. J. Immunol..

[121] Zhou Y., Sonobe Y., Akahori T., Jin S., Kawanokuchi J., Noda M., Iwakura Y., Mizuno T., Suzumura A. (2011). IL-9 promotes Th17 cell migration into the central nervous system via CC chemokine ligand-20 produced by astrocytes. J. Immunol..

[122] Purwar R., Schlapbach C., Xiao S., Kang H.S., Elyaman W., Jiang X., Jetten A.M., Khoury S.J., Fuhlbrigge R.C., Kuchroo V.K. (2012). Robust tumor immunity to melanoma mediated by interleukin-9-producing T cells. Nat. Med..

[123] Lu Y., Hong S., Li H., Park J., Hong B., Wang L., Zheng Y., Liu Z., Xu J., He J. (2012). Th9 cells promote antitumor immune responses in vivo. J. Clin. Investig..

[124] Ye Z.J., Yuan M.L., Zhou Q., Du R.H., Yang W.B., Xiong X.Z., Zhang J.C., Wu C., Qin S.M., Shi H.Z. (2012). Differentiation and recruitment of Th9 cells stimulated by pleural mesothelial cells in human Mycobacterium tuberculosis infection. PLoS One.

[125] Lu Y., Wang Q., Xue G., Bi E., Ma X., Wang A., Qian J., Dong C., Yi Q. (2018). Th9 Cells Represent a Unique Subset of CD4+ T Cells Endowed with the Ability to Eradicate Advanced Tumors. Cancer Cell.

[126] Rivera Vargas T., Humblin E., Végran F., Ghiringhelli F., Apetoh L. (2017). Th9 cells in anti-tumor immunity. Semin. Immunopathol..

[127] Ohtomo T., Kaminuma O., Yamada J., Kitamura N., Abe A., Kobayashi N., Suko M., Mori A. (2010). Eosinophils are required for the induction of bronchial hyperresponsiveness in a Th transfer model of BALB/c background. Int. Arch. Allergy Immunol..

[128] Xiao X., Fan Y., Li J., Zhang X., Lou X., Dou Y., Shi X., Lan P., Xiao Y., Minze L. (2018). Guidance of super-enhancers in regulation of IL-9 induction and airway inflammation. J. Exp. Med..

[129] Kerzerho J., Maazi H., Speak A.O., Szely N., Lombardi V., Khoo B., Geryak S., Lam J., Soroosh P., Van Snick J. (2013). Programmed cell death ligand 2 regulates TH9 differentiation and induction of chronic airway hyperreactivity. J. Allergy Clin. Immunol..

[130] Jia L., Wang Y., Li J., Li S., Zhang Y., Shen J., Tan W., Wu C. (2017). Detection of IL-9 producing T cells in the PBMCs of allergic asthmatic patients. BMC Immunol..

[131] Hoppenot D., Malakauskas K., Lavinskienė S., Bajoriūnienė I., Kalinauskaitė V., Sakalauskas R. (2015). Peripheral blood Th9 cells and eosinophil apoptosis in ashma patients. Medicina.

[132] Oh C.K., Leigh R., McLaurin K.K., Kim K., Hultquist M., Molfino N.A. (2013). A randomized, controlled trial to evaluate the effect of an anti-interleukin-9 monoclonal antibody in adults with uncontrolled asthma. Respir. Res..

[133] Saeki M., Kaminuma O., Nishimura T., Kitamura N., Mori A., Hiroi T. (2017). Th9 cells induce steroid-resistant bronchial hyperresponsiveness in mice. Allergol. Int..

[134] Percopo C.M., Brenner T.A., Ma M., Kraemer L.S., Hakeem R.M., Lee J.J., Rosenberg H.F. (2017). SiglecF+Gr1hi eosinophils are a distinct subpopulation within the lungs of allergen-challenged mice. J. Leukoc. Biol..

[135] Rosas-Ballina M., Olofsson P.S., Ochani M., Valdés-Ferrer S.I., Levine Y.A., Reardon C. (2011). Acetylcholine-synthesizing T cells relay neural signals in a vagus nerve circuit. Science.

[136] Abe A., Koyama S., Ohtomo T., Kitamura N., Kaminuma O., Mori A. (2012). Murine T cell-derived contractile activity for bronchial smooth muscle cells. Int. Arch. Allergy Immunol..

[137] Ohtomo T., Kaminuma O., Kitamura N., Suko M., Kobayashi N., Mori A. (2009). Murine Th clones confer late asthmatic response upon antigen challenge. Int. Arch. Allergy Immunol..

[138] Pelaia G., Renda T., Gallelli L., Vatrella A., Busceti M.T., Agati S., Caputi M., Cazzola M., Maselli R., Marsico S.A. (2008). Molecular mechanisms underlying airway smooth muscle contraction and proliferation: Implications for asthma. Respir. Med..

[139] Janssen L.J., Killian K. (2006). Airway smooth muscle as a target of asthma therapy: History and new directions. Respir. Res..

[140] Goto K., Chiba Y., Matsusue K., Hattori Y., Maitani Y., Sakai H., Kimura S., Misawa M. (2010). The proximal STAT6 and NF-κB sites are responsible for IL-13- and TNF-alpha-induced RhoA transcriptions in human bronchial smooth muscle cells. Pharmacol. Res..

[141] Chen H., Tliba O., Van Besien C.R., Panettieri R.A., Amrani Y. (2003). TNF-alpha modulates murine tracheal rings responsiveness to G-protein-coupled receptor agonists and KCl. J. Appl. Physiol..

[142] Nishimura T., Kaminuma O., Saeki M., Kitamura N., Matsuoka K., Yonekawa H., Mori A., Hiroi T. (2016). Essential Contribution of CD4^+^ T Cells to Antigen-Induced Nasal Hyperresponsiveness in Experimental Allergic Rhinitis. PLoS ONE.

